# Echinocystic acid alleviated hypoxic-ischemic brain damage in neonatal mice by activating the PI3K/Akt/Nrf2 signaling pathway

**DOI:** 10.3389/fphar.2023.1103265

**Published:** 2023-02-09

**Authors:** Yuan Li, Ling Chen, Da Zheng, Jian-Xia Liu, Chao Liu, Shao-Hua Qi, Peng-Chao Hu, Xiao-Fei Yang, Jia-Wei Min

**Affiliations:** ^1^ Key Laboratory of Cognitive Science, Laboratory of Membrane Ion Channels and Medicine, College of Biomedical Engineering, South-Central Minzu University, Wuhan, China; ^2^ Department of Systems Medicine and Bioengineering, Houston Methodist Cancer Center, Weill Cornell Medicine, Houston, TX, United States; ^3^ Department of Oncology, Xiangyang No. 1 People’s Hospital, Hubei University of Medicine, Xiangyang, China

**Keywords:** echinocystic acid, HIBD, OGD/R, neonatal, oxidative stress, apoptosis, PI3K/Akt/Nrf2 pathway

## Abstract

Neonatal hypoxic-ischemic encephalopathy (HIE) is considered a major cause of death and long-term neurological injury in newborns. Studies have demonstrated that oxidative stress and apoptosis play a major role in the progression of neonatal HIE. Echinocystic acid (EA), a natural plant extract, shows great antioxidant and antiapoptotic activities in various diseases. However, it has not yet been reported whether EA exerts a neuroprotective effect against neonatal HIE. Therefore, this study was undertaken to explore the neuroprotective effects and potential mechanisms of EA in neonatal HIE using *in vivo* and *in vitro* experiments. In the *in vivo* study, a hypoxic-ischemic brain damage (HIBD) model was established in neonatal mice, and EA was administered immediately after HIBD. Cerebral infarction, brain atrophy and long-term neurobehavioral deficits were measured. Hematoxylin and eosin (H&E), terminal deoxynucleotidyl transferase dUTP nick end labeling (TUNEL) and dihydroethidium (DHE) staining were performed, and the contents of malondialdehyde (MDA) and glutathione (GSH) were detected. In the *in vitro* study, an oxygen-glucose deprivation/reperfusion (OGD/R) model was employed in primary cortical neurons, and EA was introduced during OGD/R. Cell death and cellular ROS levels were determined. To illustrate the mechanism, the PI3K inhibitor LY294002 and Nrf2 inhibitor ML385 were used. The protein expression levels of p-PI3K, PI3K, p-Akt, Akt, Nrf2, NQO1, and HO-1 were measured by western blotting. The results showed that EA treatment significantly reduced cerebral infarction, attenuated neuronal injury, and improved brain atrophy and long-term neurobehavioral deficits in neonatal mice subjected to HIBD. Meanwhile, EA effectively increased the survival rate in neurons exposed to OGD/R and inhibited oxidative stress and apoptosis in both *in vivo* and *in vitro* studies. Moreover, EA activated the PI3K/Akt/Nrf2 pathway in neonatal mice following HIBD and in neurons after OGD/R. In conclusion, these results suggested that EA alleviated HIBD by ameliorating oxidative stress and apoptosis *via* activation of the PI3K/Akt/Nrf2 signaling pathway.

## 1 Introduction

Neonatal hypoxic-ischemic encephalopathy (HIE), induced by a hypoxic event and the interruption of cerebral blood supply to the brain, is one of the leading causes of mortality and lifelong disability. Neonatal HIE causes approximately 25% of neonatal deaths worldwide (Yildiz et al., 2017). Its annual incidence is 1–8 per 1,000 live births in developed countries and approximately 26 per 1,000 live births in low- and middle-income countries (Douglas-Escobar and Weiss, 2015; Millar et al., 2017). Thus far, therapeutic hypothermia (TH) represents the only reliable standard therapeutic strategy for neonates with HIE, which maintains a core body temperature of 33.5°C for 72 h commencing within 6 h of birth (Higgins et al., 2011). However, it can neither provide complete neuroprotection nor exert obvious effects on severe HIE (Zalewska et al., 2015). Hence, new effective therapies for neonatal HIE are urgently needed.

Following the hypoxia-ischemia event, reactive oxygen species (ROS) production dramatically increases and overwhelms the endogenous antioxidant system (Zhao M. et al., 2016). The large excess of ROS not only triggers oxidative stress but also results in neuronal apoptosis, both of which play a major role in the pathogenesis of neonatal HIE (Wang et al., 2022). Therefore, enhancing the body’s antioxidant capacity and thus eliminating excessive ROS may be an effective approach for ameliorating neonatal HIE.

Nuclear factor erythroid 2-related factor 2 (Nrf2) is a master regulator of the cellular antioxidant response (Wang et al., 2020). Under basal conditions, Nrf2 is maintained at a low level through targeted degradation by ubiquitylation (Kobayashi et al., 2004). Under conditions of oxidative stress, Nrf2 dissociates from Kelch-like ECH-associated protein 1 (Keap1) to translocate into the nucleus, where it binds to antioxidant response elements (AREs) and then enhances the expression of many antioxidant-encoding genes, such as NAD(P)H-ubiquinone oxidoreductase 1 (NQO1), heme oxygenase-1 (HO-1), and glutathione (GSH) (Mei et al., 2022). Then, these proteins work against ROS and confer endogenous protection against neonatal HIE (Zeng et al., 2020). Our previous study demonstrated that genistein provides neuroprotective effects against hypoxic-ischemic brain damage (HIBD) in neonatal mice *via* the Nrf2/HO-1 axis (Li Y. et al., 2022). It has also been shown that Nrf2 can be regulated by the PI3K/Akt signaling pathway, which exerts neuroprotective effects in neonatal HIE (Luo et al., 2021). Thus, searching for new compounds able to activate the PI3K/Akt/Nrf2 pathway is likely to be beneficial for treating neonatal cerebral hypoxic-ischemic injuries.

**FIGURE 1:**
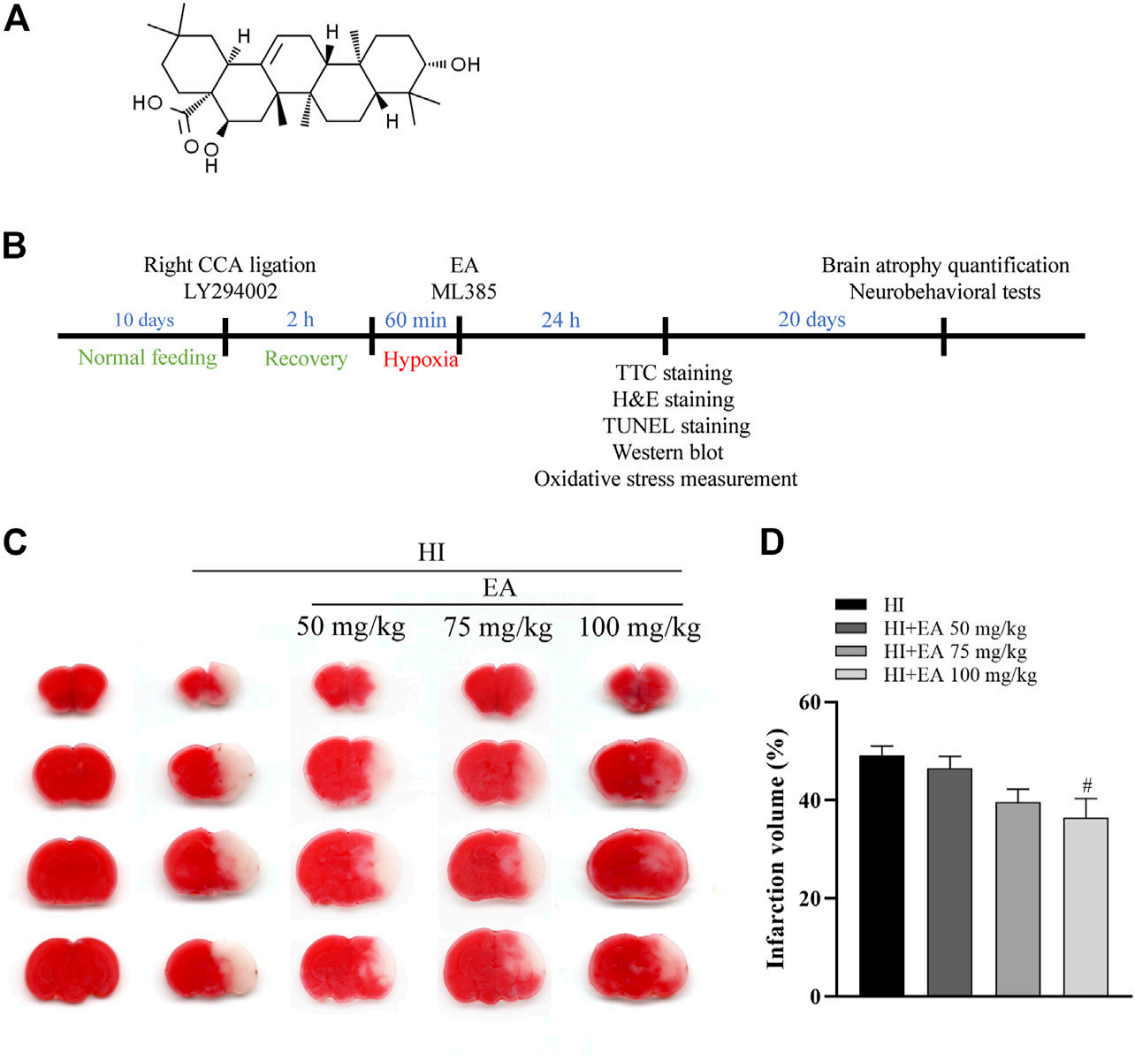
Effects of EA on cerebral infarction following HIBD. **(A)** The chemical structure of EA. **(B)** Schedule of the surgery, treatment protocols and experimental tests *in vivo* study. **(C)** Representative images of TTC-stained coronal brain sections from the sham group, HI group and groups treated with different doses of EA. **(D)** Quantitative analysis of the brain infarct volume. Data are presented as the means ± SEM, *n* = 10. ^#^ *p* < 0.05 vs. the HI group.

Echinocystic acid (EA) (Figure 1A), a pentacyclic triterpene derived from various herbs, such as *Codonopsis lanceolata* and *Acacia* (Lee et al., 2002; Jaeger et al., 2017), has a wide variety of positive effects, including anti-inflammation, antioxidant, and antiapoptotic activities (Tong et al., 2004; Joh et al., 2012; Lai and Liu, 2014). Studies have revealed that EA can penetrate the blood‒brain barrier (BBB) and exert positive effects on brain function (Park et al., 2017). Furthermore, EA provided neuroprotective effects in intracerebral hemorrhage (ICH) and Parkinson’s disease (PD) *via* the PI3K/Akt pathway (Chen et al., 2020; He et al., 2021). However, the protective effects of EA on neonatal HIE and its underlying mechanisms have not been studied.

Herein, a HIBD model was established in neonatal mice, and an oxygen-glucose deprivation/reperfusion (OGD/R) model was employed in primary cortical neurons, which were used to explore the neuroprotective effects of EA and to assess whether the PI3K/Akt/Nrf2 signaling pathway is involved in this process.

## 2 Materials and methods

### 2.1 Animals

In this study, C57BL/6 mice (RRID:MGI:2159769) were obtained from Hubei Research Center of Laboratory Animals (Wuhan, China). All C57BL/6 mice were grouped randomly and maintained in an environment with suitable humidity (55% ± 10%) and constant temperature (22°C ± 2°C). All mice had free access to standard food and water, and a 12 h light/12 h dark cycle (07:00 AM-07:00 PM) was used. All animal procedures strictly complied with the Animal Research Ethics Committee of South-Central Minzu University (No. SYXK 2016-0089) and the NIH guidelines. For the *in vivo* study, the inclusion criteria of the pups were healthy pups with reasonable body weight (4.5–5.5 g) at postnatal day 10. The exclusion criteria of the pups were dead pups occurred during or immediately after HIBD.

### 2.2 Establishment of the HIBD model

The neonatal HIBD model was performed by modification of the Rice-Vannucci Model (Zhang et al., 2021). Briefly, postnatal day 10 C57BL/6 mice (mean body weight: 5 g) were anesthetized by isoflurane (4% for inducing and 1.5%–2% for maintaining). Subsequently, a midline cervical cut (0.5 cm) was made on the neck to expose the right common carotid artery (CCA). The CCA was double-ligated with 6–0 surgical silk suture thread and cut between the two ligatures by a specific microclipper. The incision was then sutured with 5–0 surgical silk. The surgery time for each pup was maintained within 5 min. The body temperature of the pups was kept at 37°C ± 0.5°C during the operation by using a heating pad. Next, the pups were returned to their dams to recover for 2 h. After that, the mice were put in a 37°C chamber (AIPUINS, China) containing 10% oxygen and 90% nitrogen for 60 min. Finally, the pups were returned to their dams after being placed on a temperature-controlled heat pad for 15 min. Sham group mice underwent anesthesia and right common carotid artery exposure only. Pups of both genders were equally distributed among experimental groups and all analyses were performed in a blinded set-up.

### 2.3 Treatment groups and pharmacological interventions

The pups used in this study were randomly divided into the following five groups: the sham group, HI group, HI + EA group, HI + EA + LY294002 group, and HI + EA + ML385 group. For pharmacological interventions, the sham and HI groups were treated with 5% DMSO dissolved in saline. EA (Solarbio, China) was dissolved in 5% DMSO in saline and administered intraperitoneally at doses of 50 mg/kg, 75 mg/kg, and 100 mg/kg immediately after HIBD. The PI3K inhibitor LY294002 (10 μL of 20 mM in DMSO, intraperitoneally, Selleck Chemicals, United States) was injected before the surgery (Zhang et al., 2021). The Nrf2-specific inhibitor ML385 (30 mg/kg, Selleck Chemicals, United States) was dissolved in 3.5% DMSO in saline and administered intraperitoneally immediately following HIBD (Figure 1B) (Cui et al., 2021).

### 2.4 2,3,5-Triphenyltetrazolium chloride (TTC) staining

At 24 h post-HIBD, mouse brains were quickly removed and stored at −20°C for 10 min to slightly indurate the tissue. Then, the tissues were sliced into four 2 mm pieces. The sections were immersed in 2% 2,3,5-triphenyltetrazolium chloride (TTC, Sigma‒Aldrich, Germany) solution for 10 min at 37°C and fixed with 4% paraformaldehyde overnight at 4°C. Images of the stained tissues were captured by a scanner (EPSON, Japan), and the infarct area was analyzed by Image-Pro Plus 6.0 software (Image-Pro Plus, RRID:SCR_007369) (Min et al., 2019). The number of mice was ten in each group.

### 2.5 Hematoxylin and eosin (H&E) staining

Brain samples were fixed in 4% paraformaldehyde for 24 h and embedded in paraffin at 24 h after HIBD. Then, the brain tissues were coronally cut into 4 μm sections to perform H&E staining as previously described (Min et al., 2015). Images were obtained in the parietal cortex and the cornu ammonis 1 (CA1) area of the hippocampus by using a Nikon DS-U3 camera (Tokyo, Japan) with a ×40 objective. The number of mice was one in each group.

### 2.6 Terminal deoxynucleotidyl transferase dUTP nick end labeling (TUNEL) staining

The pups were sacrificed 24 h after HIBD, and TUNEL staining was performed as previously described (Li Y. et al., 2022). The TUNEL-positive cells in six randomly selected fields in the ipsilateral parietal cortex and the hippocampal CA1 region were photographed by using a Nikon DS-U3 camera with a ×40 objective. The following formula was applied to calculate the ratio of apoptotic neurons: (number of TUNEL-positive cells/number of cells) ×100%. The number of mice was three in each group.

### 2.7 Dihydroethidium (DHE) staining

The whole brains of mice were quickly collected and stored at −80°C at 24 h after HIBD, and DHE staining was performed as previously described (Li Y. et al., 2022). The images were collected in six randomly selected fields in the ipsilateral parietal cortex and the hippocampal CA1 area with a fluorescence Nikon C2 confocal microscope (Tokyo, Japan) with a ×20 objective. The number of mice was three in each group.

### 2.8 MDA and GSH measurements

At 24 h following HIBD, the animals were sacrificed, and the ipsilateral brain tissues were harvested and washed with phosphate-buffered saline (PBS). Then, the MDA content and the GSH level were detected by using the Lipid Peroxidation MDA Assay Kit (Beyotime, China) and the Glutathione Assay Kit (Beyotime, China), respectively (Li Y. et al., 2022). The number of mice was six in each group.

### 2.9 Neurobehavioral assessments

Neurobehavioral tests were performed 3 weeks after HIBD.

For the grip test, the YLS-13A Grip Strength System (Jinan Yiyan Technology Development Co., Ltd., China) was used to measure the grip strength of the contralateral forelimb in mice. Specifically, pups were suspended by their tails and approached to a 2 mm in diameter metal wire. Once the mice grasped the middle of this metal wire by their contralateral forelimb, they were gently pulled backwards horizontally until the metal wire was released. The average of three observed peak values of each mouse was used for analysis (Zhang et al., 2021).

For the corner test, the corner was composed of 2 boards (30 cm × 20 cm × 1 cm) combined at a 30° angle. The mice without training were encouraged to enter into this corner in the middle. A trail was recorded when the animals reached the corner and their vibrissae were stimulated. The ratio of right turns during 20 trials was calculated (Al Mamun et al., 2018).

For the Y maze, testing is performed by a Y-shaped maze (RWD, China) with three opaque arms oriented at 120° angles from each other. Each mouse was placed in a particular arm of the Y-shaped maze and allowed to randomly explore the three arms for 8 min. An entry was defined as all four limbs of the mouse were within an arm. A successful alternation occurs when the mouse moved into three different arms sequentially. The number of arm entries and successful alternations were traced. Spontaneous alternation (%) = [successful alternations/(the total number of arm entries −2)] ×100% (Kraeuter et al., 2019). The number of mice was nine in the sham group and was eight in the HI group and was ten in the HI + EA group.

### 2.10 Brain atrophy quantification

At 3 weeks after HIBD, the mice were sacrificed, and the brain tissues were immediately removed. The ipsilateral and contralateral hemispheres were split at the midline. Then, the separate tissues were weighed on a high-precision balance (sensitivity ±0.001 g). The following formula was used to express the percentage of brain tissue loss: (ipsilateral hemisphere weight/contralateral hemisphere weight) × 100% (Zhang et al., 2021). The number of mice was nine in the sham group and was eight in the HI group and was 10 in the HI + EA group.

### 2.11 Cultures of primary cortical neurons

Primary cortical neurons were isolated from the cerebral cortex of fetal C57BL/6 mice (embryonic 16–18 days), as described in previous studies (Zhang et al., 2018). The dissociated cortical cells were cultured in polylysine-coated 96-, 24- or 6-well plates and grown in neurobasal medium containing 2% B-27, 1% penicillin/streptomycin and 1% L-glutamine (Gibco, United States). Cultures were kept at 37°C in a 5% CO_2_ incubator, and the medium was refreshed every 3 days with 50% fresh medium. A preliminary experiment was conducted to test the potential toxic effects of EA. A stock solution of 10 mM EA was dissolved in DMSO and further diluted by the culture medium to make working solutions. The neurons were treated with different concentrations of EA (10, 15, 20, and 25 μM), and cell viability assays were performed.

### 2.12 OGD/R injury and drug treatment

For OGD/R, the neurons were incubated in glucose- and oxygen-free Dulbecco’s modified Eagle’s medium (DMEM, Gibco, United States) and placed in a hypoxic chamber (StemCell Technologies, Canada) filled with 95% N_2_ and 5% CO_2_ at 37°C. After 4 h of challenge, the cells were returned to normoxic conditions and cultured in normal complete medium for 24 h (Liu et al., 2020). Cells were divided into the following five groups: the control group, OGD/R model group, OGD/R + EA group, OGD/R + EA + LY294002 group, and OGD/R + EA + ML385 group. The control group was cultured under normal conditions. DMSO (0.2% final concentration) was used in the control and OGD/R groups. EA was used at final concentrations of 5, 10, 15, and 20 μM. LY294002 (20 μM, dissolved in 0.1% DMSO) was used as indicated (Zhao T. Z. et al., 2016). ML385 (5 μM, dissolved in 0.02% DMSO) was introduced into the cell culture medium (He et al., 2020). All drugs were maintained during OGD/R (Figure 6A).

### 2.13 Cell viability assay

At 24 h following OGD, cell viability was determined using a cell counting kit-8 (CCK-8, Beyotime, China) according to the instructions. Briefly, the neurons were incubated with 10% CCK-8 at 37°C in a 5% CO_2_ incubator for 4 h, and absorbances at 450 nm were read on a microplate reader (Thermo, United States). The number of cell cultures was eight in each group.

### 2.14 Measurement of ROS

The ROS content of the neurons was detected with a Reactive Oxygen Species Assay Kit (Beyotime, China) at 24 h after OGD. In brief, the cells were incubated with 10 μM 2′,7′-dichlorodihydrofluorescein diacetate (DCFH-DA) for 20 min at 37°C in a 5% CO_2_ incubator and then washed with DMEM 3 times. Six randomly selected fields were photographed in each sample in each group using a fluorescence Nikon C2 confocal microscope with a ×20 objective. The average fluorescence intensity of each sample in each group was evaluated using Image J (RRID:SCR_003070) and expressed as fluorescence percent (%) in comparison with control group. The number of cell cultures was four in each group.

### 2.15 Propidium iodide (PI) staining

At 24 h post-OGD, the cells were stained with PI (1:3,000, Yeason, China) for 20 min in the dark, incubated with an anti-NeuN antibody (Abcam Cat# ab104225, RRID:AB_10711153) overnight at 4°C, and then incubated with a fluorescein isothiocyanate (FITC)-conjugated anti-rabbit secondary antibody (biosharp Cat# BL033A, RRID:AB_2924943) for 2 h at room temperature. Six randomly selected fields were collected in each sample in each group by a fluorescence Nikon C2 confocal microscope with a ×20 objective. The percentage of PI-positive cells (%) = [number of PI-positive cells (red)/number of total cells (green)] ×100% (Li Y. et al., 2022). The number of cell cultures was four in each group.

### 2.16 Western blotting

Proteins extracted from the ipsilateral hemisphere collected 24 h after HIBD and from primary cortical neuron cultures 24 h after OGD were used for western blot analysis. Western blotting was performed as previously described (Zhang et al., 2021). In brief, the proteins were separated on 10% SDS−PAGE gels (Boster, China) and transferred onto polyvinylidene difluoride membranes (Millipore, United States). The membranes were blocked with 5% nonfat milk at room temperature for 1 h and then incubated at 4°C overnight with the following primary antibodies: anti-Bcl-2 (Cell Signaling Technology Cat# 3498, RRID:AB_1903907), anti-Bax (Cell Signaling Technology Cat# 2772, RRID:AB_10695870), anti-cleaved-caspase-3 (Cell Signaling Technology Cat# 9661, RRID:AB_2341188), anti-p-PI3K (Bioss Antibodies Cat# bs-6417R, RRID:AB_2924944), anti-PI3K (Bioss Antibodies Cat# bs-10657R, RRID:AB_2924945), anti-p-Akt (Cell Signaling Technology Cat# 4060, RRID:AB_2315049), anti-Akt (Cell Signaling Technology Cat# 4691, RRID:AB_915783), anti-Nrf2 (ABclonal Cat# A1244, RRID:AB_2759282), anti-NQO1 (Santa Cruz Biotechnology Cat# sc-32793, RRID:AB_628036), and anti-HO-1 (ABclonal Cat# A1346, RRID:AB_2760320), and anti-β-actin (Cell Signaling Technology Cat# 4970, RRID:AB_2223172) was used as an internal control. Subsequently, the membranes were incubated with HRP-labeled secondary antibody (biosharp Cat# BL003A, RRID:AB_2827666) for 1 h at room temperature. Finally, the visualization of the protein bands was performed using chemiluminescence (ECL, Beyotime, China) and a bioanalytical imaging system (Azure Biosystems, United States). Band density was analyzed using Quantity One 4.6.1 software (Quantity One 1-D Analysis Software, RRID:SCR_014280). The number of mice was six and the number of cell cultures was three.

### 2.17 Statistical analysis

The primary outcome for HIBD experiments was infarct volume, which was used to determine the group size for each experiment based on our preliminary experiments. All experimental data were analyzed by using GraphPad Prism 7.0 (GraphPad Prism, RRID:SCR_002798) and were presented as the means ± standard errors of the means (SEMs). For statistical analyses, one-way ANOVA followed by Tukey’s *post hoc* test was used for comparisons between multiple groups. *p* < 0.05 was considered statistically significant.

## 3 Results

### 3.1 EA decreased the infarct volume following HIBD

To explore the protective action of EA, neonatal mice were treated with different EA dosages (50 mg/kg, 75 mg/kg, and 100 mg/kg) immediately after HIBD. Compared with the HI group, the infarct volume was significantly decreased in pups treated with EA at a dose of 100 mg/kg (36.43% ± 3.964% vs. 49.15% ± 1.931%, ^#^ *p* < 0.05, *n* = 10 in each group) (Figures 1C, D). Thus, a 100 mg/kg dose of EA was used for further *in vivo* experiments. This finding revealed that EA markedly reduced HIBD-induced cerebral infarction.

### 3.2 EA attenuated neuronal damage and apoptosis following HIBD

**FIGURE 2:**
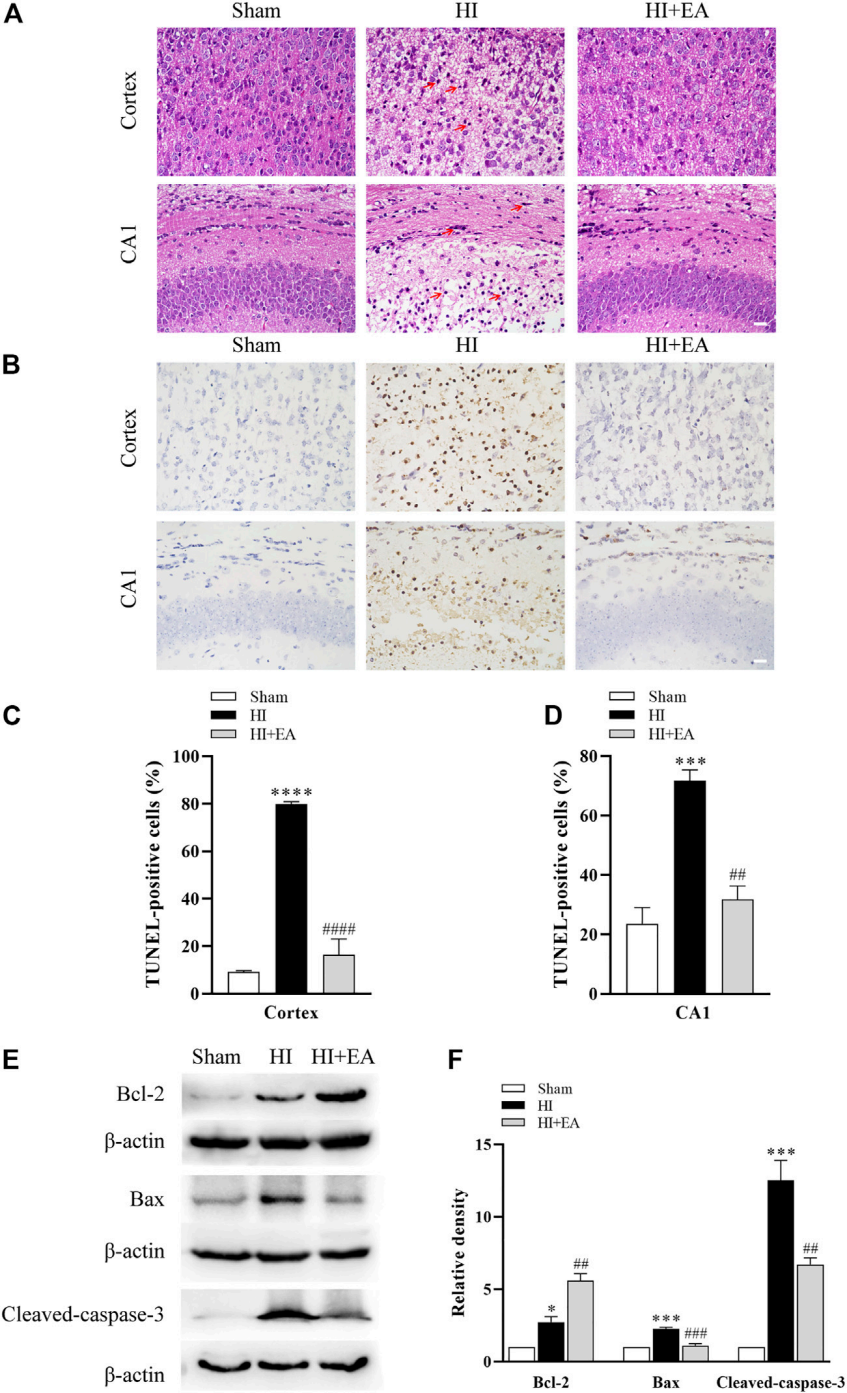
Effects of EA on neuronal damage and apoptosis following HIBD. **(A)** Representative photomicrographs of H&E staining in the ipsilateral parietal cortex and the hippocampal CA1 region (scale bar: 20 μm), *n* = 1. **(B)** Representative photomicrographs of TUNEL staining in the ipsilateral parietal cortex and the CA1 area of hippocampus (scale bar: 20 μm). **(C,D)** The percentage of apoptotic cells in the ipsilateral parietal cortex and the hippocampal CA1 area, *n* = 3. **(E)** Levels of the Bcl-2, Bax, cleaved-caspase-3 and β-actin. **(F)** Quantification of Bcl-2, Bax and cleaved-caspase-3 expressions. The red arrowheads indicate the damaged neurons. Data are presented as the means ± SEM, *n* = 3. *p < 0.05, ***p < 0.001, ****p < 0.0001 vs. the sham group; ^##^ *p* < 0.01, ^###^ *p* < 0.001, ^####^ *p* < 0.0001 vs. the HI group.

As shown in Figure 2A, neurons in the sham group (*n* = 1 in each group) were structurally integrated and regularly arranged in the ipsilateral parietal cortex and the hippocampal CA1 region. Reasonably, in the HI group, neurons exhibited atrophic structures and a disordered distribution, and the nuclei of neurons were stained and pyknotic in both cortex and the hippocampal CA1 region; however, they were partially recovered by EA treatment. TUNEL staining was used for detecting the numbers of apoptosis cells. As shown in Figures 2B–D, TUNEL staining demonstrated a large proportion of TUNEL-positive neurons in the ipsilateral parietal cortex (79.79% ± 1.111% vs. 9.186% ± 0.4997%, ****p < 0.0001, *n* = 3 in each group) and the hippocampal CA1 area (71.77% ± 3.569% vs. 23.57% ± 5.467%, ***p < 0.001) of the HI group compared to the sham group. However, compared with the HI group, neuronal apoptosis was alleviated significantly by treatment with EA in the ipsilateral parietal cortex (16.35% ± 6.679%, ^####^ *p* < 0.0001) and in the hippocampal CA1 area (31.81% ± 4.502%, ^##^ *p* < 0.01). Moreover, the western blot data in Figures 2E, F show that the level of the antiapoptotic protein Bcl-2 was obviously increased by EA treatment compared with that in the HI group (^##^ *p* < 0.01, *n* = 3 in each group). In addition, the expression levels of the proapoptotic proteins Bax (***p < 0.001) and cleaved caspase-3 (***p < 0.001) were notably increased after HIBD but were downregulated by EA treatment (^###^ *p* < 0.001; ^##^ *p* < 0.01). These data elucidated that EA relieved HIBD-induced neuronal damage and apoptosis.

### 3.3 EA inhibited oxidative stress following HIBD

**FIGURE 3:**
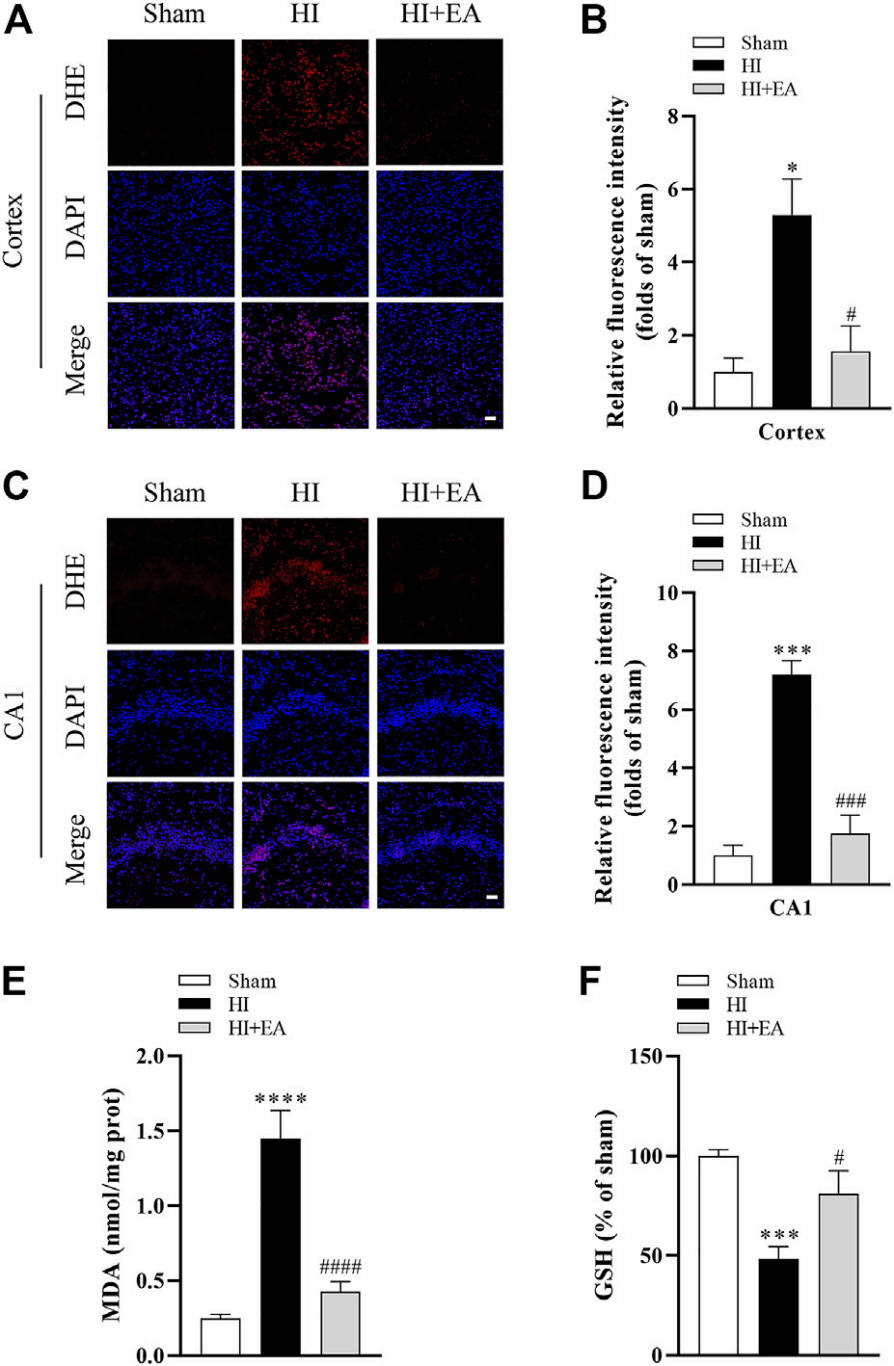
Effects of EA on oxidative stress following HIBD. **(A)** Representative pictures of DHE staining in the ipsilateral parietal cortex (scale bar: 20 μm). **(B)** Semiquantitative analysis of the DHE fluorescence intensity in the ipsilateral parietal cortex, *n* = 3. **(C)** Representative pictures of DHE staining in the CA1 area of hippocampus (scale bar: 20 μm). **(D)** Semiquantitative analysis of the DHE fluorescence intensity in the hippocampal CA1 region, *n* = 3. **(E,F)** Levels of MDA and GSH, *n* = 6. Data are presented as the means ± SEM. *p < 0.05, ***p < 0.001, ****p < 0.0001 vs. the sham group; ^#^ *p* < 0.05, ^###^ *p* < 0.001, ^####^ *p* < 0.0001 vs. the HI group.

As shown in Figures 3A–D, DHE staining revealed that the level of ROS in the ipsilateral parietal cortex and the hippocampal CA1 was low in the sham group but significantly increased in the HI group in the ipsilateral parietal cortex (*p < 0.05, *n* = 3 in each group) and in the hippocampal CA1 area (***p < 0.001). However, a marked reduction in DHE relative fluorescence intensity was observed in the HI + EA group compared with the HI group in the ipsilateral parietal cortex (^#^ *p* < 0.05) and in the hippocampal CA1 area (^###^ *p* < 0.001). In addition, the production of the lipid peroxidation product MDA was markedly increased (0.2453 ± 0.0287 nmol/mg prot vs. 1.448 ± 0.1879 nmol/mg prot, ****p < 0.0001, *n* = 6 in each group), while the antioxidant substance GSH was significantly decreased (***p < 0.001) after HIBD. However, these changes were remarkably reversed by EA treatment (^####^ *p* < 0.0001; ^#^ *p* < 0.05) (Figures 3E, F). Overall, EA restrained HIBD-induced oxidative stress in neonatal mice.

### 3.4 EA alleviated long-term neurobehavioral deficits and brain atrophy following HIBD

**FIGURE 4:**
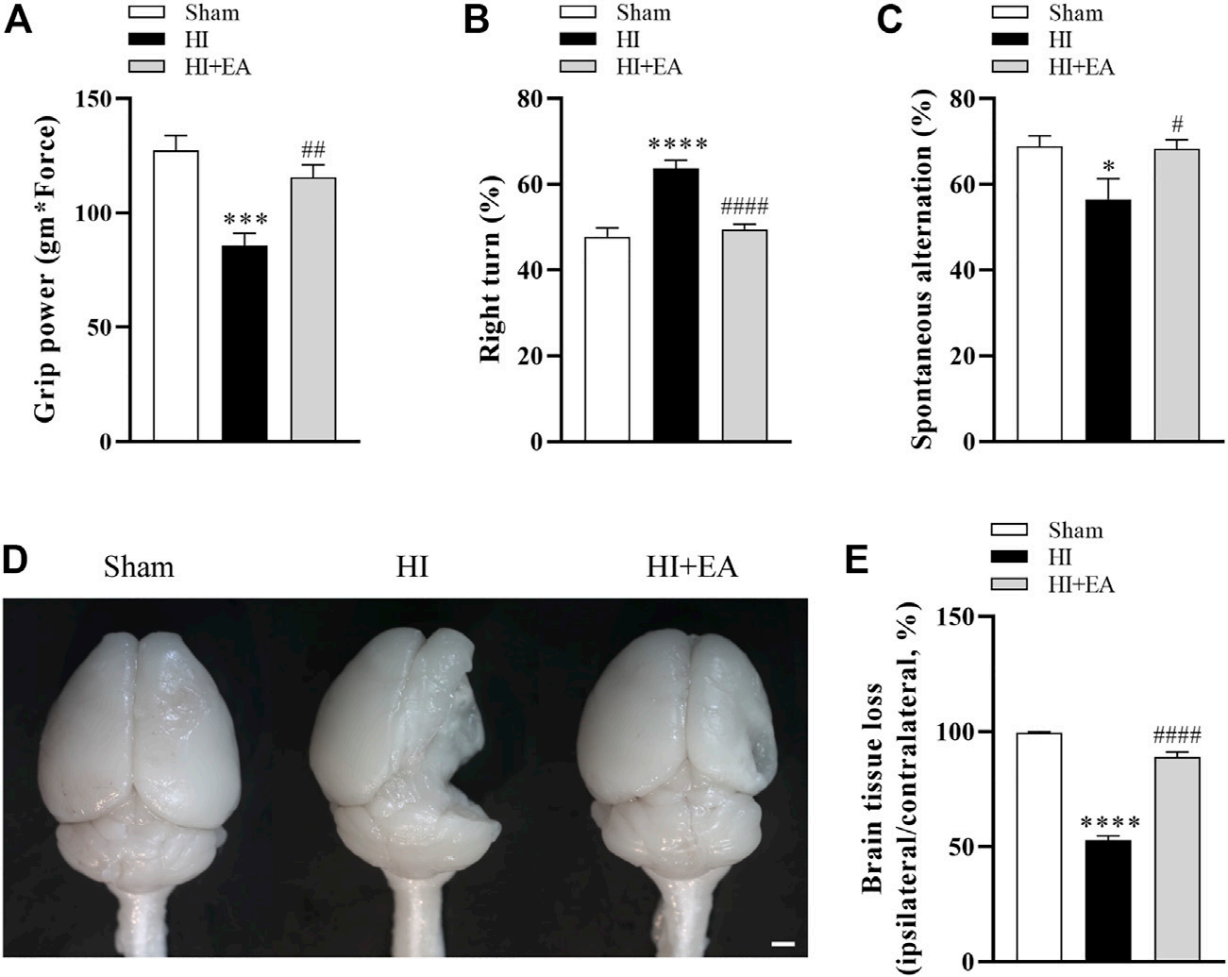
Effects of EA on long-term neurobehavioral deficits and brain atrophy following HIBD. **(A–C)** Neurological outcomes were measured by the grip strength test, the corner test, and the Y maze test (*n* = 9 in the sham group, *n* = 8 in the HI group, *n* = 10 in the HI + EA group). **(D)** Representative images of the whole brains at 21 days after HIBD (scale bar: 1 mm). **(E)** Statistics of the ratio of brain tissue loss (*n* = 9 in the sham group, *n* = 8 in the HI group, *n* = 10 in the HI + EA group). Data are presented as the means ± SEM. *p < 0.05, ***p < 0.001, ****p < 0.0001 vs. the sham group; ^#^ *p* < 0.05, ^##^ *p* < 0.01, ^####^ *p* < 0.0001 vs. the HI group.

As shown in Figures 4A–C, the administration of EA significantly attenuated long-term neurobehavioral deficits. In grip power test, the HI group (*n* = 8) was lower than the sham group (*n* = 9) (85.52 ± 5.57 gm * Force vs. 127.4 ± 6.465 gm * Force, ***p < 0.001), however, the grip strength of mice treated with EA (n = 10) was recovered effectively (115.4 ± 5.64 gm * Force, ^##^ *p* < 0.01). In corner test, the percentage of right turns was significantly increased after HI (63.75% ± 1.83% vs. 47.78% ± 2.06%, ****p < 0.0001), the EA treatment reversed this tendency obviously (49.5% ± 1.167%, ^####^ *p* < 0.0001). The ratio of spontaneous alternation in Y maze indicates the learning and memory function of pups. Mice in the HI group showed lower percentage alternations compared with the sham group (56.52% ± 4.853% vs. 68.87% ± 2.452%, *p < 0.05), which was enhanced by EA treatment notably (68.32% ± 2.055%, ^#^ *p* < 0.05). Likewise, no brain tissue loss in the ipsilateral hemisphere was observed in the sham group. However, the brain tissue loss in the HI group was markedly increased (52.84% ± 1.904% vs. 99.58% ± 0.4279%, ****p < 0.0001), which was attenuated by EA intervention (89.05% ± 2.102%, ^####^ *p* < 0.0001) (Figures 4D, E). These results showed that EA ameliorated HIBD-induced long-term neurobehavioral deficits and brain atrophy.

### 3.5 EA conferred neuroprotection against HIBD by activating the PI3K/Akt/Nrf2 signaling pathway

**FIGURE 5:**
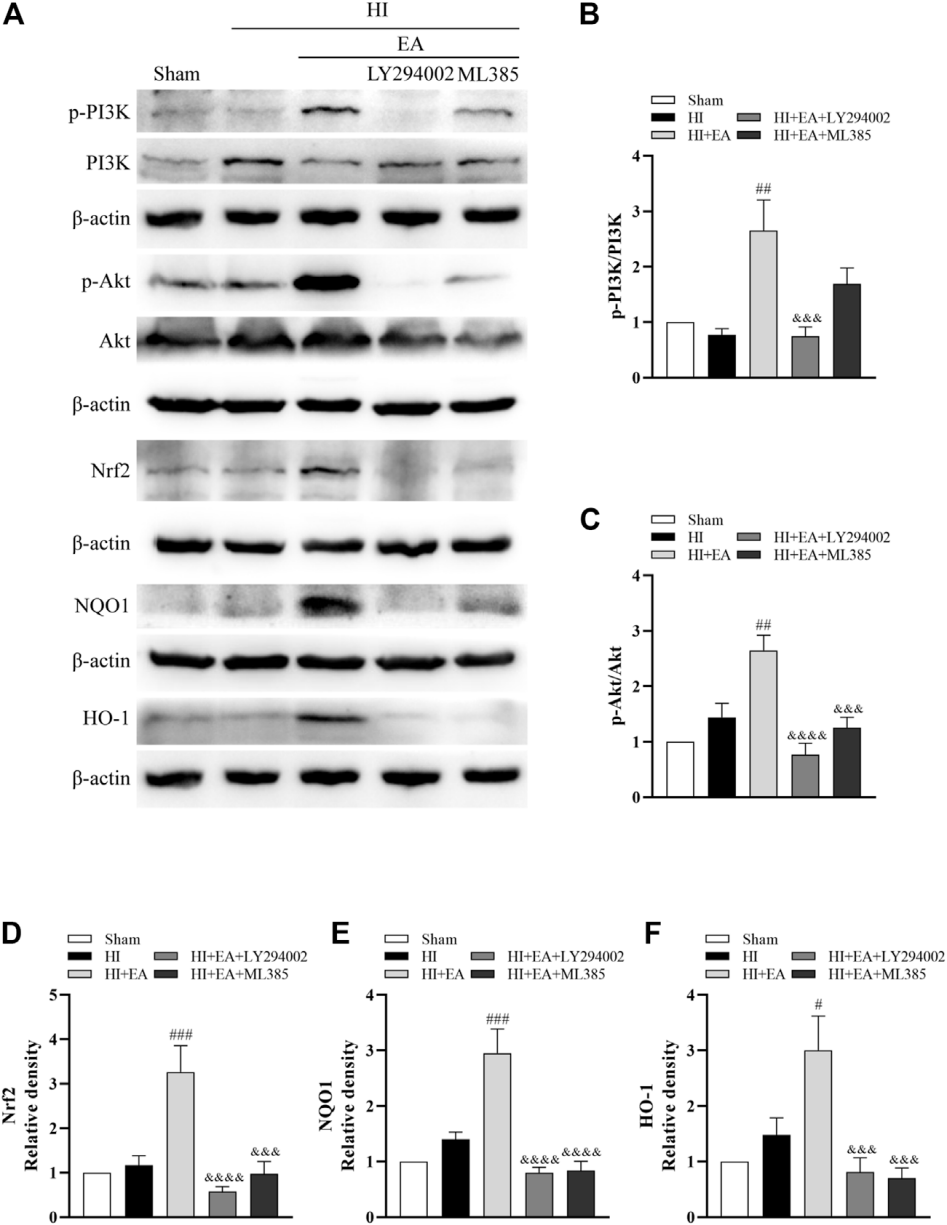
Effects of EA on the PI3K/Akt/Nrf2 signaling pathway following HIBD. **(A)** Representative western blot bands of the p-PI3K, PI3K, p-Akt, Akt, Nrf2, NQO1, HO-1 and β-actin. **(B–F)** Western blot analysis of p-PI3K, PI3K, p-Akt, Akt, Nrf2, NQO1, and HO-1, respectively. Data are presented as the means ± SEM, *n* = 6. ^#^ *p* < 0.05, ^##^ *p* < 0.01, ^###^ *p* < 0.001 vs. the HI group; ^&&&^ *p* < 0.001, ^&&&&^ *p* < 0.0001 vs. the HI + EA group.

Western blotting was used to clarify the molecular mechanism of the neuroprotective effects of EA in HIBD. We detected the levels of p-PI3K, PI3K, p-Akt, Akt, Nrf2, NQO1 and HO-1 in the ipsilateral hemisphere (Figure 5A). As shown in Figures 5B–F, the ratios of p-PI3K/PI3K (^##^ *p* < 0.01, *n* = 6 in each group) and p-Akt/Akt (^##^ *p* < 0.01) and the levels of Nrf2 (^###^ *p* < 0.001), NQO1 (^###^ *p* < 0.001) and HO-1 (^#^ *p* < 0.05) were significantly increased by EA treatment compared to the HI group. However, the PI3K inhibitor LY294002 abolished these effects of EA. Meanwhile, ML385, a specific inhibitor of Nrf2, obviously attenuated the protein expression of Nrf2 (^&&&^ *p* < 0.001), NQO1 (^&&&&^ *p* < 0.0001) and HO-1 (^&&&^ *p* < 0.001). Consequently, these data indicated that EA protected neonatal mice from HIBD by activating the PI3K/Akt/Nrf2 signaling pathway.

### 3.6 EA ameliorated OGD/R-induced injury in primary cortical neurons

**FIGURE 6:**
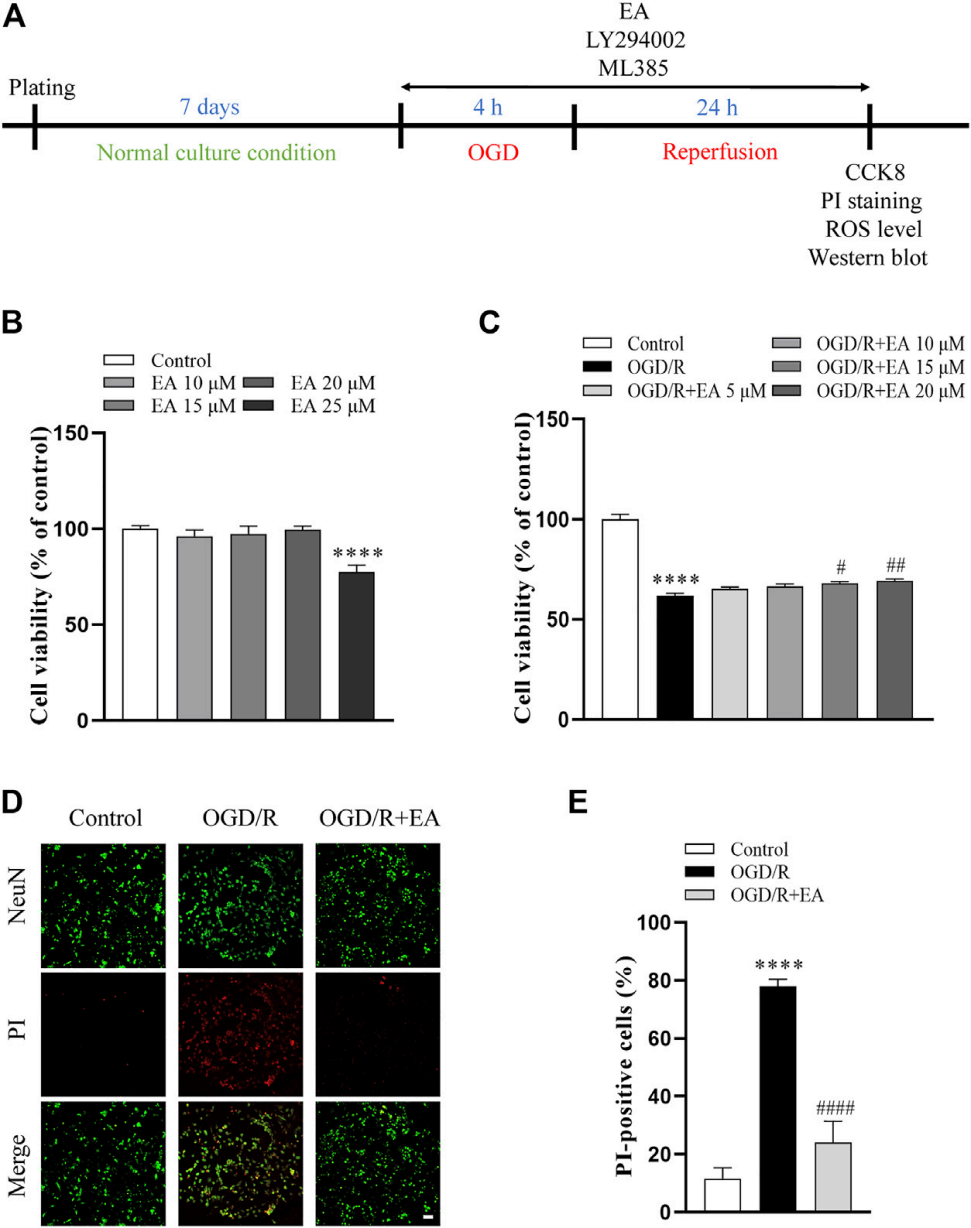
Effects of EA on OGD/R-induced neuronal injury. **(A)** Diagram of the experimental design for the *in vitro* study. **(B)** Viability of primary cortical neurons after treatment with 0, 10, 15, 20, 25 μM EA, *n* = 8. **(C)** Viability of primary cortical neurons subjected to OGD/R with or without EA (5, 10, 15, 20 μM) treatment, *n* = 8. **(D)** Representative fluorescence images of PI staining (scale bar: 20 μm). **(E)** Quantitative analysis of PI-positive cells, *n* = 4. Data are presented as the means ± SEM. ****p < 0.0001 vs. the control group; ^#^ *p* < 0.05, ^##^ *p* < 0.01, ^####^ *p* < 0.0001 vs. the OGD/R group.

CCK-8 assay was used to determine whether EA (10, 15, 20, 25 µM) has potential toxic effects on primary cortical neurons. As shown in Figure 6B, EA no more than 20 µM did not affect the viability of primary cortical neurons. Then, primary cortical neurons were exposed to OGD/R to clarify the role of EA (5, 10, 15, 20 µM) in HIBD *in vitro*. The viability of primary cortical neurons subjected to OGD/R was dramatically reduced compared with those in the control group (61.81% ± 1.303% vs. 100% ± 2.561%, ****p < 0.0001, *n* = 8 in each group) (Figure 6C). Due to the more favorable neuroprotection of EA was observed at 20 µM (69.15% ± 1.051%, ^##^ *p* < 0.01), this concentration was chosen for subsequent *in vitro* studies. PI staining was further used to identify the protective effects of EA. The results showed that the percentage of PI-positive cells was noticeably increased after OGD/R compared to the control group (77.97% ± 2.412% vs. 11.61% ± 3.707%, ****p < 0.0001, *n* = 4 in each group), but was markedly decreased by EA treatment (24.09% ± 7.271%, ^####^ *p* < 0.0001) (Figures 6D, E). These data demonstrated that EA alleviated OGD/R-induced injury in primary cortical neurons.

### 3.7 EA inhibited OGD/R-induced ROS generation in primary cortical neurons

**FIGURE 7:**
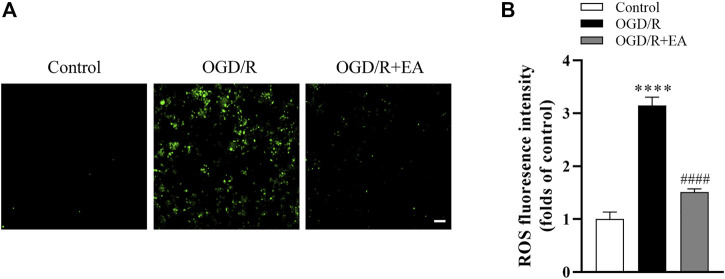
Effects of EA on OGD/R-induced ROS production. **(A)** Representative fluorescence images of ROS (scale bar: 20 μm). **(B)** ROS level analysis. Data are presented as the means ± SEM, *n* = 4. ****p < 0.0001 vs. the control group; ^####^ *p* < 0.0001 vs. the OGD/R group.

As shown in Figures 7A, B, the intracellular ROS level was measured using the DCFH-DA probe, and the ROS production in the OGD/R group was significantly increased compared to that in the control group (****p < 0.0001, *n* = 4 in each group). Interestingly, EA at a concentration of 20 µM significantly blocked this phenomenon (^####^ *p* < 0.0001), indicating that EA successfully inhibited OGD/R-induced ROS production in primary cortical neurons.

### 3.8 EA protected against OGD/R-induced neuronal injury by activating the PI3K/Akt/Nrf2 signaling pathway

**FIGURE 8:**
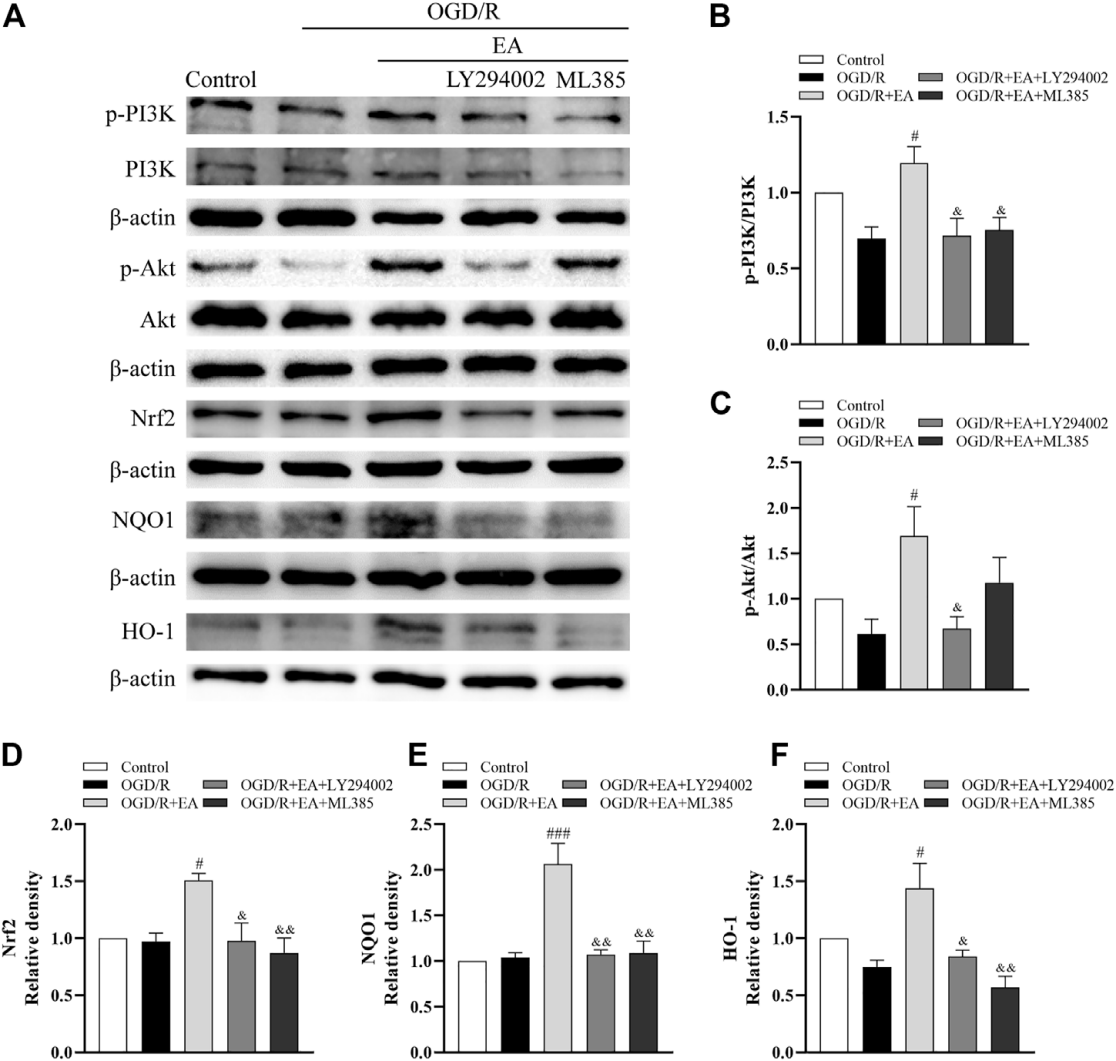
Effects of EA on the PI3K/Akt/Nrf2 signaling pathway following OGD/R. **(A)** Representative western blot bands of p-PI3K, PI3K, p-Akt, Akt, Nrf2, NQO1, HO-1 and β-actin. **(B–F)** Western blot analysis of p-PI3K, PI3K, p-Akt, Akt, Nrf2, NQO1, and HO-1, respectively. Data are presented as the means ± SEM, *n* = 3. ^#^ *p* < 0.05, ^###^ *p* < 0.001 vs. the OGD/R group; ^&^ *p* < 0.05, ^&&^ *p* < 0.01 vs. the OGD/R + EA group.

Western blot assays were also used to illuminate whether EA protects against OGD/R-induced neuronal injury by regulating the PI3K/Akt/Nrf2 signaling pathway. As indicated in Figures 8A–F, the ratios of p-PI3K/PI3K and p-Akt/Akt and the expression of Nrf2, together with the levels of its downstream proteins NQO1 and HO-1, were not altered dramatically after OGD/R. However, both of them were noticeably upregulated by EA treatment compared with the OGD/R group (phosphorylated-PI3K/PI3K: ^#^ *p* < 0.05; phosphorylated-Akt/Akt: ^#^ *p* < 0.05; Nrf2: ^#^ *p* < 0.05; NQO1: ^###^ *p* < 0.001; HO-1: ^#^ *p* < 0.05, *n* = 3 in each group). However, treatment with LY294002, an inhibitor of PI3K, mostly counteracted the effects of EA on the upregulation of both of these proteins (phosphorylated-PI3K/PI3K: ^&^ *p* < 0.05; phosphorylated-Akt/Akt: ^&^ *p* < 0.05; Nrf2: ^&^ *p* < 0.05; NQO1: ^&&^ *p* < 0.01; HO-1: ^&^ *p* < 0.05). Moreover, the increased levels of Nrf2, NQO1 and HO-1 resulting from EA treatment were suppressed by the Nrf2 inhibitor ML385 (^&&^ *p* < 0.01). We also found that EA increased the nuclear Nrf2 expression and inhibited Keap1 expression (Supplementary Figures S1, S2). These results demonstrated that EA attenuated OGD/R-induced injury in primary cortical neurons by activating the PI3K/Akt/Nrf2 signaling pathway.

## 4 Discussion

Although enormous efforts and great achievements have been made in novel therapies, effective interventions for neonatal HIE are still limited (Liu et al., 2018). In recent years, EA has been shown to exhibit excellent neuroprotection in various neurological diseases. Li et al. found that EA contributed to the reduction in the reserpine-induced pain/depression dyad in mice (Li et al., 2016). Another study showed that EA could promote neurite outgrowth in Neuro2a cells and improve cognitive function in aged mice (Park et al., 2017). Therefore, we speculate that EA has the potential for neurotherapy against neonatal HIE. In this study, firstly we found that EA decreased infarct volume, alleviated neuronal damage, and attenuated brain tissue loss and long-term neurological deficits following HIBD in pups. Secondly, EA treatment increased cell viability after OGD/R and reduced oxidative stress and apoptosis both *in vivo* and *in vitro*. Thirdly, EA exhibited neuroprotective effects on neonatal HIBD, which may be associated with the PI3K/Akt/Nrf2 signaling pathway.

A previous study reported that the infarction volume was increased significantly after HIBD (Zhang et al., 2019). Similarly, we also found obvious cerebral infarct areas in the HI group, which were then decreased by EA administration. Morphological changes in the ipsilateral brain are one of the major characteristics of neonatal HIE (Wu and Li, 2022). In the present study, we observed morphological injuries in the ipsilateral parietal cortex and the hippocampal CA1 region by H&E staining, and the results showed that EA treatment attenuated HIBD-mediated neuronal atrophy and their disordered distribution. Furthermore, a previous study found that EA alleviated memory and learning deficits in mice (Jung et al., 2012). Our results also showed that EA prevented HIBD-induced brain atrophy and long-term neurological dysfunction in neonates. These results suggested that EA reduced cerebral infarction, restrained neuronal damage and contributed to the long-term recovery of neurobehavioral deficits and brain atrophy following HIBD.

Oxidative stress and apoptosis are the major manifestations contributing to the progression of neonatal hypoxic-ischemic brain injury (Fu et al., 2021). During reperfusion, the partial recovery of oxidative metabolism results in the excessive production of ROS (Arteaga et al., 2017). It will exceed the antioxidant defense system and result in oxidative damage to proteins, lipids, and DNA, subsequently leading to cell death (Kalogeris et al., 2016). In the present study, DHE staining revealed that the production of ROS increased sharply after HIBD, which was reversed by EA treatment. Moreover, our *in vitro* data also indicated that EA treatment suppressed OGD/R-induced ROS generation in primary cortical neurons. MDA, a final breakdown product of lipid peroxidation, is used to measure the extent of oxidative damage to cell membranes (Wang et al., 2019). Our study showed that EA reduced the level of MDA in the ipsilateral hemisphere of neonatal mice following HIBD. GSH, HO-1 and NQO1 are crucial cellular antioxidants (Dinkova-Kostova and Talalay, 2010; Jian et al., 2011; Mari et al., 2020). Our *in vivo* data also showed that the contents of GSH, HO-1, and NQO1 were increased after treatment with EA. It has been demonstrated that EA can protect against cerebral ischemia/reperfusion injury through its antiapoptotic activities (Yu et al., 2019). To confirm the antiapoptotic effects of EA in neonatal HIE, TUNEL staining and PI staining were performed *in vitro* and *in vivo*, respectively. Our data indicated that EA treatment significantly restrained apoptosis, as evidenced by reduced TUNEL-positive cells and PI-positive neurons. Furthermore, the expression levels of the antiapoptotic protein Bcl-2 and proapoptotic proteins Bax and cleaved caspase-3 were also measured in this study. The ratio of Bax/Bcl-2 and the level of cleaved caspase-3 were decreased by EA treatment. Taken together, these findings indicated that EA attenuated HIBD through its antioxidative and antiapoptotic effects.

Next, we explored the underlying mechanism of EA exerting protective effects in neonatal HIBD. Nrf2 is a pivotal transcription factor known to regulate the expression of cytoprotective and antioxidant genes (Fang et al., 2020). Related research demonstrated that the upregulation of Nrf2 played a key role in the hyperbaric oxygen preconditioning-induced protection against HIBD (Zhai et al., 2016). Likewise, Gou et al. found that Nrf2-related pathways were involved in neural functional recovery after HIBD (Gou et al., 2020). The PI3K/Akt pathway is closely associated with the regulation of cell proliferation, differentiation, and apoptosis (Zhao et al., 2020). Furthermore, it has been reported that Nrf2 expression can be regulated by the PI3K/Akt pathway, which protects neonatal rats against hypoxic-ischemic brain injury (Fu et al., 2021). Studies have suggested that EA can activate the PI3K/Akt signaling pathway and thus exhibit neuroprotective effects in different diseases (Chen et al., 2020; He et al., 2021). In the present study, our western blot data showed no remarkable variations in the ratios of p-PI3K/PI3K, p-Akt/Akt or the protein levels of Nrf2, HO-1, and NQO1 after HIBD. However, EA upregulated the expression levels of the proteins mentioned above. We also found that LY294002, a PI3K inhibitor, counteracted these effects of EA. Simultaneously, the levels of Nrf2 and its downstream antioxidative proteins were significantly decreased by the Nrf2 inhibitor ML385. In addition, the abovementioned results were further confirmed by our *in vitro* studies. Taken together, EA might confer neuroprotection on neonatal HIE by activating the PI3K/Akt/Nrf2 pathway.

Some limitations existed in this study. First, the effects of LY294002 and ML385 without EA were not explored in HIBD model, since the effects of LY294002 and ML385 in HIBD model were verified by our previous study (Zhang et al., 2021) and other research (Gou et al., 2020), which demonstrated that both of these two compounds worked well in this model. However, we only detected the effects of specific inhibitors on the expression levels of PI3K/Akt/Nrf2 pathway-related proteins but did not determine whether these inhibitors could reverse the neuroprotective effects of EA *in vivo* and *in vitro*. Second, the pathogenesis of neonatal HIE is complicated; we only focused on oxidative stress and apoptosis, and other pathological factors, such as inflammation, mitochondrial dysfunction and excitotoxicity, might be involved. Finally, the expression of Nrf2 can be enhanced by the activation of silent information regulator 2-related enzyme 1 (SIRT1), ameliorating HIBD-induced ferroptosis and exerting a neuroprotective effect (Li C. et al., 2022). Whether EA can alleviate HIBD by inhibiting ferroptosis through the SIRT1/Nrf2 signaling pathway needs to be further explored.

In conclusion, the present study provided evidence that EA could alleviate hypoxic-ischemic brain injury by inhibiting oxidative stress and neuronal apoptosis by activating the PI3K/Akt/Nrf2 signaling pathway. As a natural extract from plants, EA is economical and readily available. It is expected to be further developed and utilized in clinical trials. Such as, combination of EA and therapeutic hypothermia for HIE neonates. However, we cannot ignore an important point, although the safety of EA has been widely proven, and EA has been reported in the use of food and traditional Chinese medicine in many Asian countries (Lee et al., 2002). Herbal products have been reported to cause severe side effects. Among these, liver injury, occasionally severe enough to necessitate transplantation or lead to death, has been frequently described (Stournaras and Tziomalos, 2015). So, the efficacy and safety of EA should be clearly demonstrated before it enters the markets, it is conceivable that the neonates might be more susceptible to any toxic effect of EA because of hepatic immaturity.

## Data Availability

The original contributions presented in the study are included in the article/Supplementary Material, further inquiries can be directed to the corresponding author.
