# Supplementary material for: Echinocystic acid alleviated hypoxic-ischemic brain damage in neonatal mice by activating the PI3K/Akt/Nrf2 signaling pathway
Source: Front Pharmacol. 2023 Feb 9;14:1103265. doi: 10.3389/fphar.2023.1103265 (PMC9947717; doi:10.3389/fphar.2023.1103265)
Supplement: Supplementary file 1 [file DataSheet1.pdf]

Supplementary Material

Echinocystic acid alleviated hypoxic-ischemic brain damage in neonatal mice by activating the PI3K/Akt/Nrf2 signaling pathway

Yuan Li[1†], Ling Chen[1†], Da Zheng[1], Jian-Xia Liu[1], Chao Liu[1], Shao-Hua Qi[2], Peng-Chao Hu[3], Xiao-Fei Yang[1], and Jia-Wei Min[1*]

[1]Key Laboratory of Cognitive Science, Laboratory of Membrane Ion Channels and Medicine, College of Biomedical Engineering, South-Central Minzu University, Wuhan, China

[2]Department of Systems Medicine and Bioengineering, Houston Methodist Cancer Center, Weill Cornell Medicine, Houston, USA

[3]Department of oncology, Xiangyang No.1 People's Hospital, Hubei University of Medicine, Xiangyang, China

[†]These authors contributed equally to this work

***Correspondence:** Jia-Wei Min; Key Laboratory of Cognitive Science, Laboratory of Membrane Ion Channels and Medicine, College of Biomedical Engineering, South-Central Minzu University, 182 minyuan road hongshan district Wuhan, Hubei 430074, China; Telephone: +86-027-6784-1630 E-mail: jiawei-min@hotmail.com

1 Supplemental Materials and Methods

1.1 Western blotting

Proteins extracted from primary cortical neuron cultures 24 h after OGD were used for western blotting experiments, as previously described (Zhang et al., 2021). In brief, nuclear proteins were extracted with kits (BioVision, USA) according to the instructions and total proteins were extracted with RIPA buffer (Biosharp, China). Proteins were separated in 10% SDS–PAGE gels (Boster, China) and transferred to polyvinylidene difluoride membranes (Millipore, USA). Then, the membranes were blocked in 5% nonfat milk for 1 h at room temperature and incubated overnight at 4 °C with the following primary antibodies: anti-Nrf2 (ABclonal Cat# A1244, RRID:AB_2759282), anti-Keap1 (Cell Signaling Technology Cat# 8047, RRID:AB_10860776), anti-Histone H3 (Abcam Cat# ab1791, RRID:AB_302613), and anti-β-actin (Cell Signaling Technology Cat# 4970, RRID:AB_2223172). After that, the membranes were incubated with HRP-labeled secondary antibody (biosharp Cat# BL003A, RRID:AB_2827666) for 1 h at room temperature. Finally, the visualization of the protein bands was performed using chemiluminescence (ECL, Beyotime, China) and a bioanalytical imaging system (Azure Biosystems, USA). Band density was analyzed using Quantity One 4.6.1 software (Quantity One 1-D Analysis Software, RRID:SCR_014280). The number of cell cultures was 3 in each group.

2 Supplementary Figures

S-1A

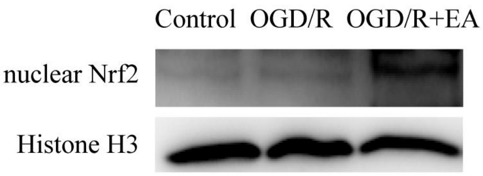

S-1B

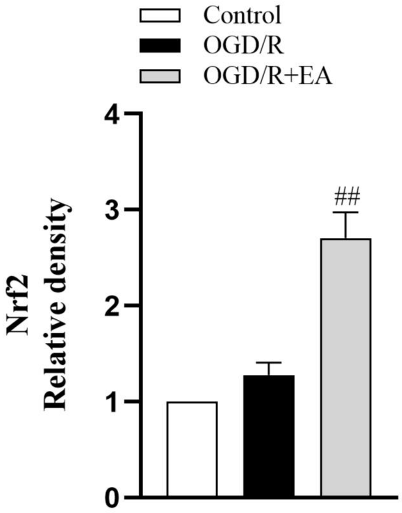

Supplementary Figure S-1. Effects of EA on the nuclear transcription of Nrf2 following OGD/R. **S-1A.** Representative western blot bands of nuclear Nrf2 and Histone H3. **S-1B.** Quantitation of **S-1A.** Data are presented as the means ± SEM, n=3. $^{\#\#}P < 0.01$ vs. the OGD/R group.

S-2A

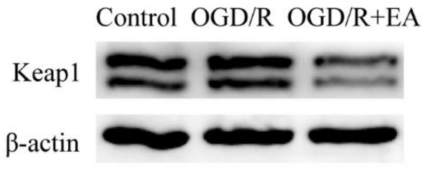

S-2B

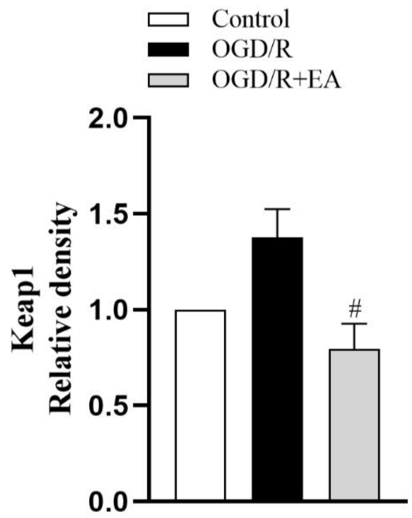

Supplementary Figure S-2. Effects of EA on the expression level of Keap1 following OGD/R. **S-2A.** Representative western blot bands of Keap1 and β-actin. **S-2B.** Quantitation of **S-2A.** Data are presented as the means ± SEM, n=3. $^{\#}P < 0.05$ vs. the OGD/R group.

3 References:

Zhang, J.J., Li, Y., Chen, S., Yang, X.F., and Min, J.W. (2021). Biphalin, a dimeric opioid peptide, reduces neonatal hypoxia-ischemia brain injury in mice by the activation of PI3K/Akt signaling pathway. *J Chem Neuroanat* 115**,** 101967. doi: 10.1016/j.jchemneu.2021.101967.
